# Human Ad19a/64 HERV-W Vaccines Uncover Immunosuppression Domain-Dependent T-Cell Response Differences in Inbred Mice

**DOI:** 10.3390/ijms24129972

**Published:** 2023-06-09

**Authors:** Isabella Skandorff, Emeline Ragonnaud, Jasmin Gille, Anne-Marie Andersson, Silke Schrödel, Lara Duvnjak, Louise Turner, Christian Thirion, Ralf Wagner, Peter Johannes Holst

**Affiliations:** 1 Department of Immunology and Microbiology, University of Copenhagen, Blegdamsvej 3B, 2200 Copenhagen, Denmark; isa@inprother.com (I.S.); lara.duvnjak@sund.ku.dk (L.D.); lturner@sund.ku.dk (L.T.); 2 InProTher, COBIS, Ole Maaloesvej 3, 2200 Copenhagen, Denmark; era@inprother.com (E.R.); aca@inprother.com (A.-M.A.); 3 Department of Biomedical Sciences, University of Copenhagen, Blegdamsvej 3B, 2200 Copenhagen, Denmark; 4 Institute of Medical Microbiology and Hygiene, Molecular Microbiology, University of Regensburg, 93053 Regensburg, Germany; jasmin.gille@klinik.uni-regensburg.de (J.G.); ralf.wagner@klinik.uni-regensburg.de (R.W.); 5 Sirion Biotech GmbH, Am Haag 6, 82166 Graefelfing, Germany; schroedel@sirion-biotech.de (S.S.); thirion@sirion-biotech.de (C.T.)

**Keywords:** adenovirus vector, human endogenous retrovirus type W (HERV-W), immunosuppressive domain, Syncytin-1

## Abstract

Expression of human endogenous retrovirus type W (HERV-W) has been linked to cancer, making HERV-W antigens potential targets for therapeutic cancer vaccines. In a previous study, we effectively treated established tumours in mice by using adenoviral-vectored vaccines targeting the murine endogenous retrovirus envelope and group-specific antigen (Gag) of melanoma-associated retrovirus (MelARV) in combination with anti-PD-1. To break the immunological tolerance to MelARV, we mutated the immunosuppressive domain (ISD) of the MelARV envelope. However, reports on the immunogenicity of the HERV-W envelope, Syncytin-1, and its ISD are conflicting. To identify the most effective HERV-W cancer vaccine candidate, we evaluated the immunogenicity of vaccines encoding either the wild-type or mutated HERV-W envelope ISD in vitro and in vivo. Here, we show that the wild-type HERV-W vaccine generated higher activation of murine antigen-presenting cells and higher specific T-cell responses than the ISD-mutated counterpart. We also found that the wild-type HERV-W vaccine was sufficient to increase the probability of survival in mice subjected to HERV-W envelope-expressing tumours compared to a control vaccine. These findings provide the foundation for developing a therapeutic cancer vaccine targeting HERV-W-positive cancers in humans.

## 1. Introduction

The emergence of cancer immunotherapeutic strategies such as immune checkpoint inhibitors (ICI) has been revolutionary in that these have shown extraordinary efficacy in certain cancer types with pre-existing T-cell responses. An alternative immunotherapeutic strategy is therapeutic cancer vaccines. Cancer vaccines can induce strong, long-lasting cellular and humoral responses toward tumour antigens, but the outcomes of the early clinical trials were disappointing (reviewed in [1]). Importantly, technological advancements within the genetic characterization of cancers and vaccine delivery systems offer new potential for the development of cancer vaccines [1], as well as clinical benefits for patients [2].

One of the expanding vaccine delivery technologies is virus-like particle (VLP)-based vaccines. VLPs consist of repetitive viral antigens assembled into small non-infectious particles, and this repetitive and structured antigen presentation makes VLP-based vaccines highly immunogenic, while still safe and well-tolerated [3,4]. Inside our bodies, VLPs are phagocytosed by antigen-presenting cells (APCs) that facilitate humoral responses by MHC class II (MHC-II)-dependent antigen presentation to CD4^+^ T-cells [3]. However, the induction of robust CD8^+^ T-cell responses relies on cross-presentation of the engulfed antigen on MHC class I (MHC-I).

Virus-like-vaccines (VLVs) encode VLP components in a viral vector, enabling classic VLP-based MHC-II antigen presentation and B-cell activation as well as direct intracellular antigen presentation via MHC-I [5]. Furthermore, this technology resolves the production and stability issues that classic VLP-based vaccines face [4], as only the vector is produced in vitro [5]. Our previous VLV studies targeting human immunodeficiency virus type 1 (HIV-1) and the murine melanoma-associated retrovirus (MelARV) utilized replication-deficient adenoviral vectors encoding structural (group-specific antigen, Gag) and transmembrane envelope (Env) proteins to produce VLPs from infected cells in vivo [6,7,8]. We are now exploring the potential of VLV vaccines encoding tumour antigens originating from human endogenous retroviruses (HERVs) and the efficacy of these vaccines in inducing immune responses to target HERV-expressing cancers.

HERV provirus genes originate from infections of the germline cells of our ancestors from now-extinct exogenous retroviruses [9]. HERVs make up almost 8% of the human genome, but due to large truncations and nonsense mutations, the vast majority of the HERV genes are today pseudogenes, and only a few open reading frames are intact [10,11]. Furthermore, HERVs are typically silenced in healthy somatic cells [12]. On the contrary, numerous reports of HERV transcription and translation across a wide spectrum of cancers and other pathologies exist [13]. Both HERV-specific cellular and humoral responses have been detected in cancer patients [14,15], indicating an incomplete tolerance to these autoantigens. In support of this, a very recent study found that many lung cancer patients exhibit humoral responses to the Env (Env K102) of one of the HERV families, HERV-K [16]. This study showed that higher transcriptional levels of Env K102 prior to treatment with ICI correlated with better treatment prognosis and overall survival following ICI treatment. Moreover, increases in HERV-K Env antibody responses correlated with post-treatment survival. Collectively, these data suggest that immune responses to HERVs can enhance tumour sensitization to cancer immunotherapy [16].

Another HERV family that has been associated with cancer malignancy is HERV-W (reviewed in [17,18]). The HERV-W genes are widely spread in the human genome, but only one HERV-W gene, *ERVWE1*, encodes a functional Env protein, Syncytin-1 [19]. Syncytin-1 has been co-opted to play important roles in human placentation, such as the cell–cell fusion of cytotrophoblasts [20,21]. Besides its roles in placentation, Syncytin-1 has been found to be expressed in different human cancer tissues and cancer cell lines (reviewed in [17,18,22]), and Syncytin-1 has been suggested to be involved in cell–cell fusions in cancers such as colorectal cancer [22,23,24]. Cell–cell fusion is also a prominent feature of bladder cancer in vitro, contributing to immune evasive properties [25], and interestingly, a study reported expression-promoting mutations in the Syncytin-1 3′-LTR in ~85% of tissue samples from patients with bladder cancer [26]. While the exact function of Syncytin-1 and other HERV-W gene products in cancer malignancy is still unclear, the tumour-associated expression makes HERV-W antigens potential targets for cancer vaccines.

Retrovirus Env proteins are immunosuppressive, and in 1985, Cianciolo et al. assigned this function to a well-conserved part of the Env protein, named the CKS-17 peptide [27]. The immunosuppressive effects of the CKS-17 peptide included shifting Th1 to Th2 cytokine responses of PBMCs and suppression of IL-2-dependent T-cell proliferation [27,28,29]. Since then, much research has aimed to determine the biological relevance of the CKS-17 peptide, specifically in relation to human and non-human endogenous retroviruses (collectively called (H)ERVs). The immunosuppressive properties of CKS-17 in (H)ERVs gained support from research performed by the group of Thierry Heidmann. They identified a minimal immunosuppressive domain (ISD) in the Env of (H)ERVs consisting of 20 amino acids, including the CKS-17 sequence [30], which across different (H)ERVs, suppressed allogeneic tumour rejection in mice [30,31,32,33]. Furthermore, they found that by amino acid substitution of position 14 and 20 of the Env ISD, the immunosuppressive function was abrogated without affecting the fusogenic and infectious activity of (H)ERV virions [33].

As mentioned, we recently generated a cancer vaccine targeting the tumour-antigens of the murine endogenous retrovirus MelARV [6,7]. To achieve the most immune stimulative vaccine, we assessed the immunogenicity of non-replicative adenoviral vectored vaccines encoding the MelARV Gag and Env proteins with (ISDmut) and without (ISDwt) mutating the ISD. We found that the MelARV ISDmut vaccine induced significantly higher immune responses and showed improved anti-cancer efficacy compared to the MelARV ISDwt vaccine [7], supporting the “ISD hypothesis” and indicating that a similar approach could be pursued for a vaccine targeting HERV-W in human cancers.

However, the ISD of the HERV-W Env Syncytin-1 is unusual. In contrast to other (H)ERVs, Mangeney et al. found that the ISD of Syncytin-1 is not able to suppress allogeneic tumour rejection, but this immunosuppressive capability could be achieved by exchanging amino acids 14 and 20 of the Syncytin-1 ISD with the corresponding amino acids of the related Mason–Pfizer monkey virus, without affecting Syncytin-1′s fusogenicity [30].

Although the findings by Mangeney et al. indicated that Syncytin-1 is not immunosuppressive [30], there have been reports of both immunosuppressive [20,34,35] and immune stimulative [36] effects of Syncytin-1. Furthermore, a study by Eksmond et al. generally questioned the immunosuppressive capacity of (H)ERV ISDs, as they could not reproduce findings by Thierry Heidmann’s group, which showed that mutating the ISD did not change virion infectivity but just immunogenicity [33]. Instead, Eksmond et al. found that mutating the ISD greatly changed infectivity, particularly in cells expressing the native cellular (H)ERV receptor [37]. This controversy highlights that the mechanism of (H)ERV ISDs is not fully understood. A recent study proposed a direct role of the (H)ERV ISD, as they found that an ISD peptide from the HERV family, HERV-H, exhibited immunosuppressive properties and direct antagonistic effects on the potassium channel KCa3.1, which is normally required for the maturation of APC and T-cell responses [38]. However, it is yet unclear how this ISD peptide would interact with KCa3.1 when part of a full-length Env protein.

In this current study, we aimed to develop a vaccine targeting HERV-W-positive human cancers using a VLV-based vaccine encoding HERV-W Gag and the HERV-W Env, Syncytin-1. To obtain the most immune stimulatory HERV-W vaccine, we assessed the immunogenicity of vaccines containing the full-length wild-type Env protein (least immunosuppressive, ISDwt) or the reverse Env (putative immunosuppressive, ISDrev). By exploring the ISD immunogenicity differences in mice lacking the native HERV-W Env cellular receptors and by using a replication-deficient adenoviral vaccine vector, we provide the optimal settings to address the conundrum of the immunological function of the ISD in full-length Env’s. In support of the original study by Mangeney et al. [30], we show that the ISDwt HERV-W vaccine induces higher activation of murine antigen-presenting cells and higher specific cellular immune responses than the HERV-W ISDrev vaccine. Finally, we show that compared to a control vaccine, the most immune stimulatory HERV-W ISDwt vaccine significantly increases survival probability in mice bearing HERV-W Env^+^ tumours.

## 2. Results

### 2.1. Design and Characterization of hAd19a/64-HERV-W Vaccines

**Figure 1:**
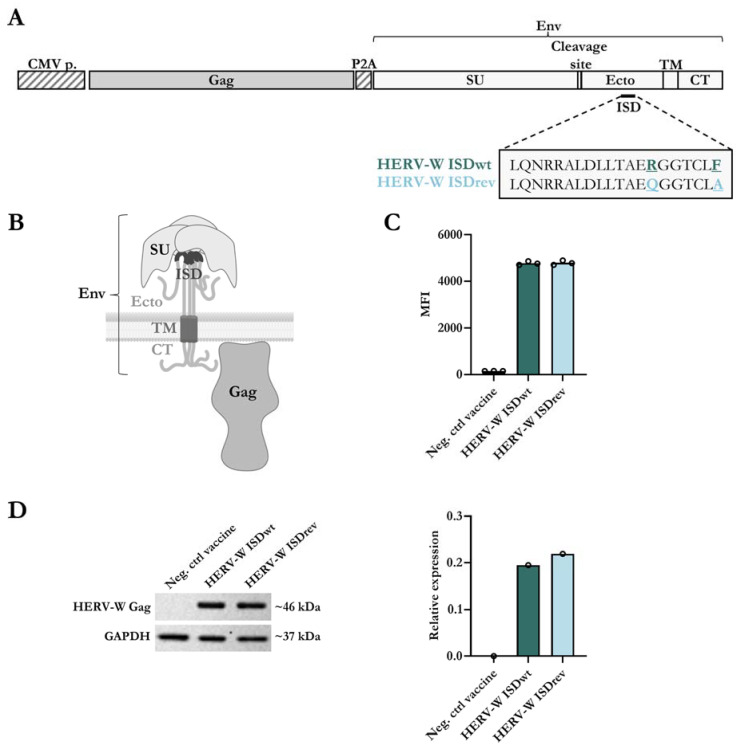
Design and characterization of the hAd19a/64-HERV-W vaccines. (**A**) Schematic representation of HERV-W vaccine antigens encoded in the replication-deficient hAd19a/64 vector, under control of a CMV promoter (CMV p.). The vaccines encode an assembled Gag sequence and the Env sequence of Syncytin-1, separated by a self-cleavable peptide (P2A). The Env protein compromises a surface subunit (SU) and a transmembrane subunit (TU). The TU is divided into an ectodomain (Ecto) with the immunosuppressive domain (ISD), a transmembrane anchor (TM), and a cytoplasmic tail (CT). The 20 amino acid sequence of the ISD is highlighted with two amino acids differences between the HERV-W ISDwt vaccine and the HERV-W ISDrev vaccine: R > Q and F > A are underlined. (**B**) Visual representation of a trimeric transmembrane HERV-W Env protein and the membrane HERV-W Gag protein, created using BioRender.com. (**C**) Level of Env expression on the surface of A549 cells, measured by flow cytometry 24 h post transduction with 10MOI of either an empty hAd19a/64 vaccine (Neg. ctrl vaccine), hAd19a/64 HERV-W ISDwt, or hAd19a/64 HERV-W ISDrev. The bar graph illustrates the geometric mean fluorescent intensity (MFI) of the Env protein. Each dot represents one technical replicate. (**D**) Gag expression was detected by western blot from cell lysates 24 h after transduction of A549 cells with 50MOI of either the Neg. ctrl vaccine, HERV-W ISDwt, or HERV-W ISDrev. As a loading control, the housekeeping protein, GAPDH, was also measured. Left panel shows the western blot membrane with the expected protein sizes (kDa). Right panel shows the relative expression differences of Gag and GAPDH proteins, calculated using the iBright analysis software V5.1.0.

We generated two HERV-W vaccines to assess the immunogenicity of the ISD: one vaccine containing the wild-type HERV-W Env sequence of Syncytin-1 (HERV-W ISDwt); and one vaccine encoding this Env sequence but with two mutations in the ISD (R > Q and F > A) (HERV-W ISDrev), as originally defined by Mangeney et al. (Figure 1A) [30]. Both vaccines were designed to potentially obtain Gag-protein based VLPs covered with the Env protein on the surface. To explore this property, we encoded the Env sequences together with an assembled sequence of HERV-W Gag [39] separated by a self-cleavable P2A peptide sequence. The full-length Syncytin-1 Env (from now HERV-W Env) sequence consists of two subunits: the surface subunit (SU); and the transmembrane subunit (TU), which contains an ectodomain (Ecto), a transmembrane region (TM), and a cytoplasmic tail (CT) (Figure 1A,B). The HERV-W vaccines were incorporated into a replication-deficient human adenoviral vector type 19a/64 (hAd19a/64) under control of a CMV promoter. The expression level of the vaccine antigens was evaluated in vitro in the human A549 cells, which are easily transduced by the hAd19a/64 vector. Similar levels of Env surface proteins were expressed by both vaccines as determined by flow cytometry (Figure 1C, Supplementary Figure S1A,B). In addition, comparable expression levels of intracellular Gag protein were confirmed by western blot of cell lysates (Figure 1D). Furthermore, the ISD mutations did not impair the HERV-W Env fusion activity in human T24 cells (Supplementary Figure S1C), implying that the main mechanical fusion activity of the Env was not altered by the ISD mutations, as previously described by Mangeney et al. [30].

We next assessed the ability of the vaccines to generate VLPs in transduced A549 cells. We did not detect Gag and Env proteins in cell supernatants. We also did not observe any extracellular VLPs by TEM (Supplementary Figure S1D,E). This result was surprising because VLPs were previously observed using vaccines encoding Env and Gag of MelARV [6,7] and HERV-K (unpublished results, patent no. WO2019043127) (Supplementary Figure S1E), and because of the reported association of the used HERV-W Gag sequence with the formation of particles in patients with multiple sclerosis [39,40]. Instead, we observed a few VLP-like structures in intracellular vesicles that were smaller (~60–70 nm) and less electron-dense than the VLPs produced by the MelARV and HERV-K vaccines (~100 nm) (Supplementary Figure S1E). These results suggest that the HERV-W vaccine produced VLPs but without their extracellular secretion.

### 2.2. HERV-W ISDrev Vaccine Affects the Activation of Mature BMDCs

**Figure 2:**
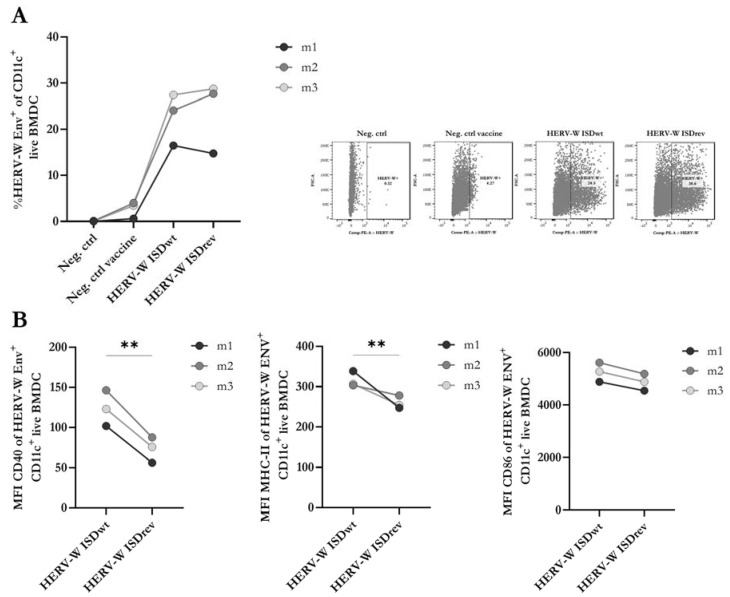
Analysis of activation markers and HERV-W Env on transduced BMDCs. Flow cytometry of matured bone-marrow-derived dendritic cells (BMDCs) isolated from BALB/c mice (*n* = 3) and transduced with either the empty hAd19a/64 vector (Neg. ctrl vaccine), the HERV-W ISDwt vaccine, or the HERV-W ISDrev vaccine at 1000MOI, 24 h prior to analysis. (**A**) The graph to the left depicts the percentage of HERV-W Env expressing BMDCs out of CD11c^+^ live cells. The right panel of dot plots illustrates the most representative gates of HERV-W Env^+^ cells. (**B**) Geometric mean fluorescent intensity (MFI) of activation markers on HERV-W Env^+^ CD11c^+^ live BMDCs. The graphs show the MFI of CD40, MHCII, and CD86 arranged from left to right. The bullets depict the mean of three technical replicates per mouse (m1-3). Lines link BMDCs transduced with either of the HERV-W vaccines from the same mouse. Expression differences were calculated using a two-tailed paired *t*-test and statistical significance is indicated with asterisks; ** = *p* < 0.01.

In our recent study of a vaccine targeting MelARV, we showed that inhibition of the ISD function, by two point-mutations, enhanced the expression of activation markers (CD40 and MHC-II) on the surface of transduced bone marrow-derived dendritic cells (BMDCs) [7]. To investigate whether a similar effect could be observed with the HERV-W ISDwt vaccine in comparison to the HERV-W ISDrev, we analyzed the levels of three activation markers (MHC-II, CD86, and CD40) on the surface of mature BMDCs from three different mice (m1, m2, and m3) by flow cytometry 24 h after virus transduction. Non-transduced BMDCs (Neg. ctrl) and BMDCs transduced with an empty hAd19a/64-vectored vaccine (Neg. ctrl vaccine) were used as negative controls. Both HERV-W vaccines appeared similar on the flow cytometry forward- and side-scatter and induced similar levels (~20%) of Env expression on the surface of transduced BMDCs (Figure 2A, Supplementary Figure S2A,B) without affecting the viability (Supplementary Figure S2C). Out of the total live BMDCs (HERV-W Env +/−), both HERV-W vaccines reduced the surface expression of CD40 and, to a lesser extent, MHC-II, while CD86 remained unchanged (Supplementary Figure S2D). However, within the HERV-W Env^+^ BMDC population, the HERV-W ISDwt vaccine induced significantly higher expression of CD40 and MHC-II than the HERV-W ISDrev (Figure 2B), as well as a higher percentage of CD40-expressing cells (Supplementary Figure S2E). The expression level of CD86 remained unchanged (Figure 2B, Supplementary Figure S2A,E). These findings suggest that the HERV-W ISDrev vaccine inhibits the immunostimulatory capacity of antigen-expressing BMDCs compared to the HERV-W ISDwt vaccine.

As the point mutations in the ISD of the HERV-W ISDrev vaccine affected the expression of activation markers on the surface of BMDCs, we decided to investigate the potential impact on the production of pro-inflammatory cytokines and chemokines. We screened a panel of 10 proinflammatory biomarkers, where four were selected for further analysis. The levels of these biomarkers in the supernatant of BMDCs were measured 24 h after transduction (Supplementary Figure S2F). We observed a strong increase in the levels of TNFα and IL-12p70 following transduction with both HERV-W vaccines, compared to the empty vector control, but no significant differences between the two HERV-W vaccines (Supplementary Figure S2F). The relatively low transduction efficacy of mouse cells with the hAd19a/64 vector in vitro may have masked any differences between the activity of these vaccines in the matured BMDCs.

### 2.3. HERV-W ISDwt Induces Higher Env-Specific T-Cell Responses Than HERV-W ISDrev

**Figure 3:**
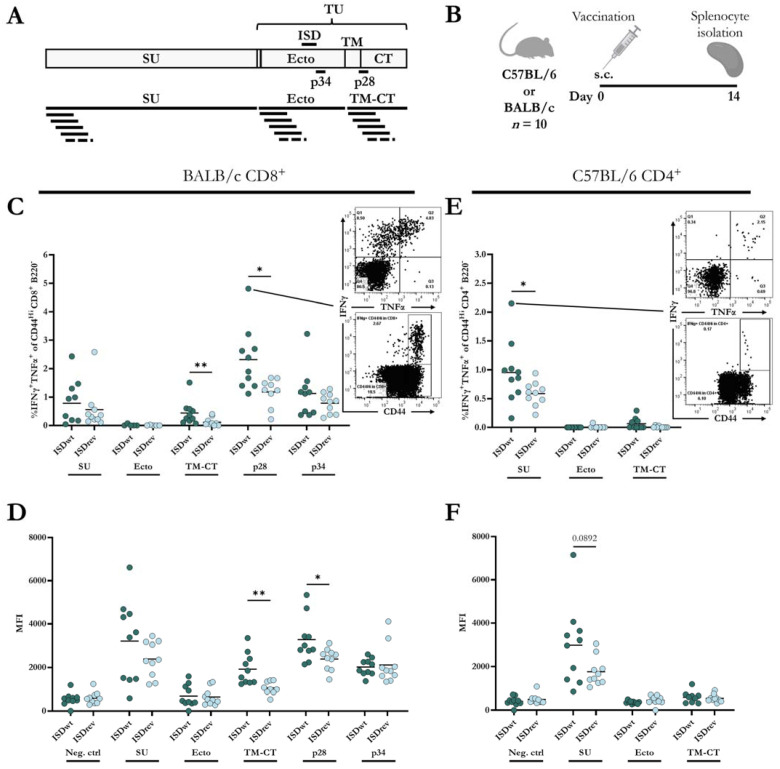
Cellular immune responses to HERV-W vaccines. (**A**) Graphical depiction of HERV-W Env domains, visualizing the regions covered by the single peptides 28 (p28) and 34 (p34). The bottom of the illustration shows peptide pools containing 16-mer peptides overlapping with 11 amino acids. (**B**) BALB/c (*n* = 10) and C57BL/6 (*n* = 10) mice were vaccinated subcutaneously (s.c.) with either HERV-W ISDwt or HERV-W ISDrev. Splenocytes were isolated 14 days later and stimulated with peptides in (**A**) to measure IFNγ-and TNFα-producing T-cells. (**C**,**E**) Percentage of IFNγ and TNFα out of CD44^Hi^, CD8^+^ B220^−^ cell population measured in BALB/c (**C**) and C57BL/6 (**E**). (**D**–**F**) Geometric mean fluorescent intensity (MFI) of IFNγ^+^ out of IFNγ^+^, CD44^Hi^, CD8^+^ B220^−^ cells, measured in BALB/c (**D**) and C57BL/6 (**F**). Each bullet represents one mouse, and the bars indicate the mean. One outlier was removed in group “BALB/c ISDwt p28”. Response differences were calculated by a non-parametric, two-tailed Mann–Whitney test, and significance levels are indicated by asterisks; * = *p* < 0.05 and ** = *p* < 0.01.

To our knowledge, there are no reports on the characterization of HERV-W-specific T-cell responses in mice, meaning that no T-cell epitopes have been predicted or validated in mice. Furthermore, there is no clear consensus on the use of overlapping peptide pools to measure both CD8^+^ and CD4^+^ T-cell responses [41,42,43]. Therefore, we examined T-cell responses to the HERV-W vaccines in two different mouse species (BALB/c and C57BL/6) using 8- and 9-mer predicted peptides of HERV-W Gag and Env, as well as 16-mer and 20-mer peptides spanning the whole Env sequence with 11 or 15 amino acids overlap, respectively. The overlapping peptides were divided into three pools covering the Env’s SU, Ecto, or TM and CT region (Figure 3A). Using the online MHC-I prediction tool, NetMHC-4.0, we selected 35 of the top predicted peptides for BALB/c MHC haplotypes and 16 for C57BL/6 MHC haplotypes [44,45]. These peptides were assessed for their ability to induce IFNγ and TNFα CD8^+^ T-cell responses 14 days after immunization with the HERV-W ISDwt vaccine. Only vaccinated BALB/c mice showed CD8^+^ T-cell responses to two peptides: peptide 28 (p28, FGPCIFNLL) from the Env TM-CT region and peptide 34 (p34, CYYVNQSGI) from the Env Ecto-region. No Gag-specific T-cell responses were observed.

Next, we examined the T-cell responses to the HERV-W ISDwt and ISDrev vaccines using the two predicted peptides and the 16- and 20-mer overlapping peptide pools in BALB/c and C57BL/6 mice. T-cell responses were measured by intracellular staining of splenocytes from mice 14 days after vaccination (Figure 3B, Supplementary Figure S3A). The two vaccines induced comparable frequencies of IFNγ- and TNFα-producing CD8^+^ and CD4^+^ T-cells. The responses were generally higher using the 16-mer overlapping peptide pools than the 20-mer, apart from the CD4^+^ responses to the Ecto-pool (Supplementary Figure S3B). Thus, we decided to evaluate the T-cell responses using only the 16-mer overlapping peptide pools and the two validated 9-mer epitopes.

In BALB/c mice, the HERV-W ISDwt vaccine induced a significantly higher frequency of IFNγ and TNFα-producing CD8^+^ T-cells to p28 of the TM-CT region (Figure 3C), as well as a higher intensity (MFI) (Figure 3D, Supplementary Figure S3C), and calculated total number of IFNγ-producing CD8^+^ T-cells compared to the HERV-W ISDrev vaccine (Supplementary Figure S3D). Although a difference in the magnitude of the responses, the vaccine response difference was also observed towards the TM-CT peptide pool (Figure 3C,D, Supplementary Figure S3D), strengthening the use of peptide pools to screen T-cell responses.

CD8^+^ T cell responses were also induced towards the peptide pools of the SU domain and the single peptide p34 of the Ecto-region but without significant differences between the two vaccines (Figure 3C,D, Supplementary Figure S3D). Both vaccines, however, exhibited poor CD4^+^ T-cell responses in BALB/c mice (Supplementary Figure S3E–G).

In C57BL/6 mice, the HERV-W ISDwt vaccines induced a higher frequency of IFNγ-and TNFα-producing CD4^+^ T-cells to the SU peptide pool compared to the ISDrev vaccine (Figure 3E). The IFNγ MFI was also slightly higher (Figure 3F), but not the total number of IFNγ-producing CD4^+^ T-cells (Supplementary Figure S3I). In contrast to BALB/c mice, both vaccines generated very poor CD8^+^ T-cell responses in C57BL/6 mice (Supplementary Figure S3H,J,K).

The CD8^+^ and CD4^+^ T-cell responses to the Ecto-pool containing the ISD were minor in both BALB/c and C57BL/6 (Figure 3C,E, Supplementary Figure S3F,J), but as mentioned, BALB/c mice responded to p34 from the Ecto-region at similar CD8^+^ T-cell levels in the two vaccine groups (Figure 3C).

Overall, both vaccines induced CD8^+^ and CD4^+^ T-cell responses to different epitopes of the Env protein of HERV-W in two different mouse species (BALB/c and C57BL/6). The mutations in the ISD of HERV-W ISDrev vaccine significantly reduced the frequency and functionality of CD8^+^ T-cell responses to the TM-CT region and CD4^+^ T-cell responses to the SU region in BALB/c and C57BL/6 mice, respectively.

### 2.4. Humoral Responses and Vaccine Efficacy against a Murine Cancer Model Expressing HERV-W Env

**Figure 4:**
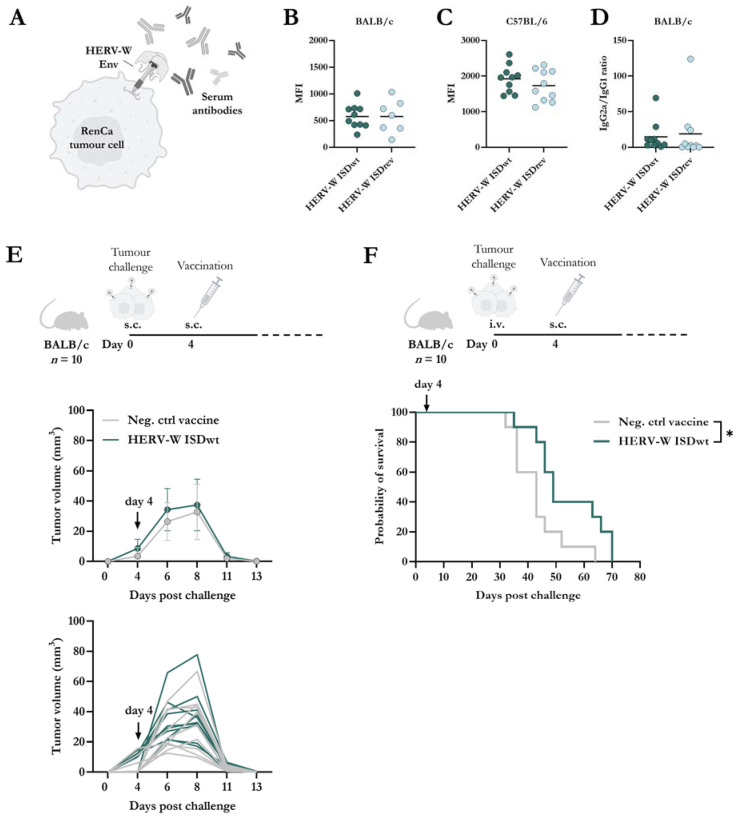
Antibody responses and therapeutic vaccine efficacy against HERV-W Env-expressing RenCa cells. (**A**) Graphical illustration of the binding of antibodies to HERV-W Env protein on the surface of a RenCa tumour cell (HERV-W Env^+^ RenCa), created using BioRender.com. HERV-W Env^+^ RenCa cells were stained with serum from BALB/c (**B**) or C57BL/6 (**C**) mice, 14 days after vaccination with either HERV-W ISDwt or HERV-W ISDrev (*n* = 10). Serum binding was analyzed by flow cytometry. (**B**,**C**) Geometric mean fluorescent intensity (MFI) of serum IgG binding to HERV-W Env^+^ RenCa cells. MFI was evaluated from live cells with each sample run in duplicates. Each bullet represents the mean of the duplicates for each mouse, and the line shows the mean. The results of three mice were excluded from the ISDrev group due to high background in the pre-immune serum. Response differences were calculated by a non-parametric two-tailed Mann–Whitney test. (**D**) Total HERV-W-specific IgG2a/IgG1 isotype ratio of serum from the vaccinated BALB/c mice. Each bullet represents the mean of the duplicates for each mouse, and the line shows the mean of the group. (**E**,**F**) BALB/c mice were challenged either subcutaneously (s.c.) (**E**) or intravenously (i.v.) (**F**) with 0.5 × 10^6^ HERV-W Env^+^ RenCa cells on day zero. On day 4, mice were immunized with 2 × 10^7^ infectious units of either the control vaccine (Neg. ctrl vaccine) or the HERV-W ISDwt vaccine (*n* = 10). For s.c. injection, tumour growth was measured with a calliper over a period of 14 days (**E**), while for the i.v. injection, mice were euthanized when reaching humane endpoints, and tumour nodules in the lungs were evaluated (**F**). (**E**) Bullets indicate the mean and whiskers show the standard deviation. Lines without whiskers and bullets illustrate the mean. (**F**) Grey lines represent mice vaccinated with the negative control vaccine, and green lines represent HERV-W ISDwt-vaccinated mice. Arrows indicate the day of vaccination. Statistical survival difference was analyzed by a log-rank test (Mantel-Cox), * = *p* < 0.05.

In Mangeney et al.’s study, the HERV-W ISDwt protein induced higher IgG responses to a recombinant trimeric Ecto-region of ISDwt compared to the ISDrev protein in Swiss mice immunized twice and with sera collected 11 days after the first immunization [30]. We next wanted to evaluate whether similar humoral responses could be observed between the HERV-W ISDwt and ISDrev vaccines. However, in this present study, we were interested in the humoral responses targeting the full-length native Env protein and not just the Ecto-region. For that purpose, serum was collected 14 days after vaccination of BALB/c and C57BL/6 mice. To measure serum responses to the natively folded HERV-W Env protein, we generated a murine RenCa cell line constitutively expressing the full Env protein (RenCa-HERV-W Env) using lentivirus transduction (Figure 4A). The long-term surface expression of the Env protein was confirmed by flow cytometry with a HERV-W SU-binding antibody (Supplementary Figure S4A,B). We then tested the binding capacity of the serum isolated from vaccinated mice to the RenCa-HERV-W Env cancer cell line. Both HERV-W vaccines induced comparable levels of HERV-W Env-specific IgG antibody responses in BALB/c and C57BL/6 mice (Figure 4B,C, Supplementary Figure S4C,D).

Given that the IgG responses were similar in both vaccine groups, we sought to investigate if any disparities in responses could be observed in the IgG isotype profiles. IgG2a isotype responses are classic markers of Th1 responses, whereas IgG1 is for Th2 responses in BALB/c mice [46]. However, the IgG2a/IgG1 response ratio to HERV-W Env SU was indistinguishable between the two vaccine groups in BALB/c mice (Figure 4D).

Following, we wanted to explore whether immune responses induced by a HERV-W vaccine could induce tumour protection. Since the activation of mouse BMDCs and HERV-W Env-specific T-cell responses were higher with the HERV-W ISDwt vaccine compared to ISDrev, we proceeded with the HERV-W ISDwt vaccine. BALB/c mice *(n* = 10) were challenged either locally in the flank (subcutaneous (s.c.) injection) or distantly in the lung (intravenous (i.v.) injection) with the RenCa-HERV-W Env cancer cell line. Mice were then vaccinated either with the HERV-W ISDwt vaccine or a control vaccine, four days after the tumour challenge (Figure 3E,F). All mice challenged locally developed palpable tumours, but these were eliminated rapidly. By day 13 after the tumour challenge, the tumours were undetectable in both groups, possibly due to vaccine-independent T-cell responses directed towards the foreign HERV-W Env protein expressed by the cancer cells, but the vaccine was not required for this (Figure 4E). In the distantly spread tumour model, mice were euthanized upon reaching human endpoints, and tumour nodules in the lungs were evaluated. Although nodules were observed in all mice, those vaccinated with the HERV-W ISDwt vaccine exhibited a significantly higher survival rate compared to the control vaccine (Figure 4F).

## 3. Discussion

We generated two hAd19a/64-vectored HERV-W vaccines that encode a HERV-W Gag sequence and included either the wild-type Env sequence of Syncytin-1 (ISDwt) or the postulated less immunogenic version, ISDrev [30]. Both vaccines induced the secretion of proinflammatory cytokines from mature BMDCs in vitro and Env-specific T- and B-cell responses in mice. Expression levels of CD40 and MHC-II on mature BMDCs decreased by transduction with either of the HERV-W vaccines but with the lowest levels observed in ISDrev-expressing cells. This suggests that the ISDrev mutations can negatively affect immune activation responses in APCs cells. Additionally, the ISDrev vaccine induced lower CD8^+^ T-cell responses to the TM-CT region in BALB/c mice and lower CD4^+^ T-cell responses to the SU region in C57BL/6 mice, than the ISDwt vaccine. Overall, these findings agree with the conclusions of the original study of the HERV-W ISD [30]. However, these immune response differences were not reflected in the antibody responses to the native full-length Env protein, as measured by flow cytometry, or in the IgG isotype profiles, as measured by ELISA. Finally, after finding HERV-W ISDwt to be the most immunogenic vaccine, we showed an increase in survival of mice vaccinated four days following i.v. challenge with RenCa cells expressing the HERV-W Env.

Since the publication of the CKS-17 peptide in 1985, the function of retroviral Env ISDs has been a topic of debate [27]. Schlecht-Louf et al. studied the effects of two point-mutations in the ISD of the endogenous retrovirus Friend murine leukaemia virus (F-MuLV), which did not affect the “mechanical” functions of the F-MuLV virions such as fusion and virus infectivity. Unlike the F-MuLV ISDwt, the viral propagation of the F-MuLV ISDmut was eliminated in immunocompetent mice by innate and adaptive immune responses. Thus, Schlect-Louf et al. concluded that a functional ISD is required to induce Env-mediated immunosuppression in vivo [33].

Later Eksmond et al. re-examined the F-MuLV ISDmut and on the contrary, they found that the two point-mutations introduced in the ISD greatly affected the infectivity and propagation of the F-MuLV virus when produced in cells expressing the ecotropic cellular Env receptor of F-MuLV, murine cationic amino acid transporter-1 (mCAT1). However, if the virus was produced in a human cell line lacking mCAT1 expression, as in Schlecht-Loufs’ study, then the infectivity and propagation were similar between ISDwt and ISDmut F-MuLV virions. Thus, Eksmond et al. concluded that the differences in viremia in mice in Schlect-Louf et al.’s studies were more likely explained by differences in virus propagation—caused by structural fragility of the ISDmut when encountering its cellular receptor—rather than differences in immunosuppression [37]. The differences in virus propagation measured in Eksmond et al. could explain some of the outcomes in Schlecht-Louf’s study, but they do not explain the ISD-dependent immune response differences observed in our recent study on MelARV [7] and in this present study on HERV-W. While Schlecht-Louf et al. and Eksmond et al. studied infectious and propagation-competent F-MuLV virions, Daradoumis et al. used a non-replicative hAd19a/64 virus vector which only encoded a Gag and Env sequence, forming VLPs incapable of propagation [7]. The use of non-replicating particles facilitated a clear evaluation of the immune function of the ISD devoid of any confounding effects in propagation.

Eksmond et al. hypothesized that the mutations in the ISD affected the strength of the association between the SU and TU regions [37]. We cannot rule out that mutation of the ISD can affect the structure and stability of the Env. However, in Daradoumis et al.’s study, the MelARV ISDwt and ISDmut vaccines induced similar differences in levels of surface-expressed Env for murine BMDC cells and human A549 cells, although the latter presumably does not express an ecotropic cellular MelARV Env receptor [7]. In this present study, there were no differences in HERV-W Env surface expression levels between ISDwt and ISDrev in human cells (A549) nor in murine cells (BMDC), but we still observed differences in the vaccine-induced immune responses.

This present study examined the impact of ISD mutations on the induction of T-cell responses to various parts of the HERV-W Env sequence. We found that the HERV-W ISDwt vaccine generated significantly higher CD8^+^ and CD4^+^ T-cell responses to certain regions of the Env protein than the HERV-W ISDrev vaccine, indicating that the effects of the mutations are not solely structural or sequence dependent. While our study did not uncover the immune regulatory mechanisms of the ISD or address the potential direct involvement with the KCa3.1 potassium channels reported by Laska et al. [38], it does support the previously published study by Mangeney et al. and the general “ISD hypothesis” [30].

However, in contrast to Mangeney et al.’s study [30], we did not observe differences in total serum IgG responses to the cell surface expressed HERV-W Env protein, nor differences in IgG isotype ratios by ELISA. It is likely, that this could be due to differences in the experimental setups between these two studies, with the biggest difference being the presence or absence of the Env SU region. We measured serum IgG responses to the native full-length Env protein expressed on the surface of cancer cells using flow cytometry, and since the SU sequence is the same in both of our vaccines, and it is the most distal and accessible part of the protein, it is possible that serum antibodies primarily bound the SU, masking any differences in responses to the Ecto-region. While Mangeney et al. adjusted for sequence differences in the ISD peptides in ELISA by measuring response differences both to the wild-type and mutated Ecto-region [30], we still cannot rule out the possibility that measuring responses to a different Env-region impacted our results.

Based on successful VLV studies targeting HIV-1 and MelARV [6,7,8], the vaccines in this study were designed to encode the HERV-W Gag and Env sequences and showed to generate both cellular and humoral responses. Following cell entry of the adenoviral vaccines, Gag and Env serve as antigens to be directly presented on MHC-I and, through the generation of VLPs, to be presented on MHC-II and MHC-I (cross-presentation) following their uptake by professional APCs. The *ERVWE1* gene coding for Syncytin-1 is one of the few examples of HERV genes that can still produce functional proteins. Although more than 70 HERV-W Gag gene sequences have been identified in the human genome, none of these contains complete open reading frames [19]. However, HERV-W Gag RNA has been observed in non-pathological testis and placental tissue as well as in samples from patients with multiple sclerosis [47,48,49]. In addition, there are reports of endogenous HERV-W VLPs in samples from this patient group [39,40,50].

In this study, we decided to encode the longest assembled HERV-W Gag sequence that has been reported [39]. Although the Gag protein expression was confirmed by western blotting, using lysates from transduced A549 cells, we did not detect any extracellular VLPs by TEM. However, we did observe a few VLP-like structures in intracellular vesicles, but determining whether these are truly HERV-W VLPs will require good monoclonal antibodies, which are currently not available. It is possible that the encoded assembled HERV-W Gag sequence was insufficient to make fully functional Gag proteins, which is necessary for the formation of extracellular VLPs. If this is indeed the case, then the vaccine design does not constitute a fully functional VLV. Nonetheless, the inclusion of the Gag sequence still provides T-cell epitopes that can be valuable for responses against HERV-W-expressing cancers. Further research should investigate if other antigen carriers can increase antibody responses and break humoral immune tolerance in humans.

## 4. Materials and Methods

### 4.1. Cell Lines

Human A549 (ATCC, CCL-185) cells, human epithelial urinary bladder cancer cell line T24 (HTB-4; ATCC, Manassas, VA, USA), and murine renal cortical adenocarcinoma cell line (RenCa) (CRL-2947; ATCC, Manassas, VA, USA) were cultured in Ham’s F12 Nutrient Mix GlutaMAX media (Thermo Scientific™, Waltham, MA, USA), DMEM GlutaMAX, and RPMI 1640 GlutaMAX, respectively. All cell media were supplemented with 10% FBS, 100 units/mL penicillin-streptomycin (pen/strep) (Thermo Scientific™, Waltham, MA, USA), and 1 mM sodium pyruvate (Thermo Scientific™, Waltham, MA, USA).

### 4.2. Vaccine Design

The HERV-W Env genetic sequence was based on the full-length protein sequence of Syncytin-1 (*ERVWE1*, locus 7q21.2). This sequence corresponds to the Env ISDwt sequence, whereas the Env ISDrev sequence differs by two amino acids (in bold) in the ISD (underlined): R > Q and F > A, as originally described by Mangeney et al. [30].

HERV-W Env sequence:

MALPYHIFLFTVLLPSFTLTAPPPCRCMTSSSPYQEFLWRMQRPGNIDAPSYRSLSKGTPTFTAHTHMPRNCYHSATLCMHANTHYWTGKMINPSCPGGLGVTVCWTYFTQTGMSDGGGVQDQAREKHVKEVISQLTRVHGTSSPYKGLDLSKLHETLRTHTRLVSLFNTTLTGLHEVSAQNPTNCWICLPLNFRPYVSIPVPEQWNNFSTEINTTSVLVGPLVSNLEITHTSNLTCVKFSNTTYTTNSQCIRWVTPPTQIVCLPSGIFFVCGTSAYRCLNGSSESMCFLSFLVPPMTIYTEQDLYSYVISKPRNKRVPILPFVIGAGVLGALGTGIGGITTSTQFYYKLSQELNGDMERVADSLVTLQDQLNSLAAVVLQNRRALDLLTAE**R**GGTCL**F**LGEECCYYVNQSGIVTEKVKEIRDRIQRRAEELRNTGPWGLLSQWMPWILPFLGPLAAIILLLLFGPCIFNLLVNFVSSRIEAVKLQMEPKMQSKTKIYRRPLDRPASPRSDVNDIKGTPPEEISAAQPLLRPNSAGSS

As there is no full-length proviral HERV-W Gag sequence in the human genome, we used an assembled sequence of HERV-W Gag. This sequence was extracted from three HERV-W/multiple sclerosis-associated retrovirus Gag sequences—LB15 (AF123880), CL2 (AF123881), and CL17 (AF12388)—where two were extracted from cultured particles from the plasma of patients with multiple sclerosis [39]. By comparison with the “HERV17” provirus consensus sequence described by Grandi et al. [51], this HERV-W sequence encompasses a potential full-length matrix sequence and a near-full-length capsid region but lacks the nucleocapsid region [19].

HERV-W Gag sequence:

MGNVPPEAKMPLERILENWDQCDTQTLRKKRFIFFCSTAWPQYPLQGRETWLPEGSINYNIILQLDLFCRKEGKWSEVPYVQTFFSLRDNSQLCKKCGLCPTGSPQSPPPYPSVPSPTPSSTNKDPPLTQTVQKEIDKGVNNEPKSANIPRLCPLQAVRGGEFGPARVPVPFSLSDLKQIKIDLGKFSDNPDGYIDVLQGLGQSFDLTWRDIMLLLNQTLTPNERSAAVTAAREFGDLWYLSQANNRMTTEERTTPTGQQAVPSVDPHWDTESEHGDWCHKHLLTCVLEGLRKTRKKPMNYSMMSTITQGKEENLTAFLDRLREALRKHTSLSPDSIEGQLILKDKFITQSAADIRKNFKSLPLGSEQNLETLLNLATSVFYNRDQEEQAE

The HERV-W Gag and Env sequences were separated by a self-cleavable P2A peptide sequence and encoded in a replication-deficient human adenoviral vector type 19a/64 (hAd19a/64) under the control of a CMV promoter. The negative control (neg. ctrl vaccine) was based on the same adenoviral vector without antigen insert. The three adenovirus vaccines used in this paper are abbreviated in the following way: HERV-W ISDwt: hAd19a/64-CMV(TetO)-coHERV-W-ISDwt-P2TS, HERV-W ISDrev: hAd19a/64-CMV(TetO)-coHERV-W-ISDrev-P2TS, and neg. ctrl vaccine: hAd19a/64-CMV(TetO)-P2TS.

### 4.3. Adenoviral Vector Production

Plasmids encoding the antigens were synthesized by GenScript Biotech (Piscataway, NJ, USA), and the hAd19a/64 vaccines were produced as previously described in [7]. The vaccine production and validation were carried out by Sirion Biotech and InProTher Aps. In brief, the HERV-W antigens were cloned into a shuttle vector that was amplified in *E. coli* and recombined into a BAC vector containing a replication-deficient hAd19a/64 backbone (with deletion of *E1* and *E3* genes), as described by Ruzsics et al. [52]. The resulting BAC-DNA construct was linearized and transfected into a modified HEK293 production cell line. In these cells, the virus constructs were further amplified to a large-scale lysate, from which viruses were purified. Next, the DNA was isolated and sequenced for quality control. Finally, viruses were tittered in parallel by immunohistochemical detection of the adenoviral hexon protein.

### 4.4. Evaluation of Env Expression by Flow Cytometry

A549 cells were transduced with the multiplicity of infection (MOI) of 10 for the relevant hAd19a/64 vaccine. Twenty-four hours later, cells were incubated with 15 µg/mL of primary rabbit anti-human HERV polyclonal antibody (PA5-22819; Invitrogen™, Waltham, MA, USA) in FACS buffer (PBS containing 1% BSA and 0.1% NaN_3_) for 1 h at 4 °C. This antibody targets the HERV-W Env SU region. Next, cells were stained with secondary phycoerythrin (PE) donkey anti-rabbit IgG antibody (406421; BioLegend^®^, San Diego, CA, USA; 1:100) and eBioscience™ Fixable Viability Dye eFlour™ 780 (65-0865; Invitrogen™ Waltham, MA, USA; 1:1000) for 30 min at 4 °C. Finally, cells were fixated in 1% paraformaldehyde (PFA) for 15 min at 4 °C. Flow cytometry was run on the LSRFortessa™ 3-laser cell analyser (BD Biosciences, Franklin Lakes, NJ, USA), and the data were analysed using FlowJo™ v10 analysis software and GraphPad Prism 9.

### 4.5. Evaluation of Gag Expression by Western Blotting

A549 cells were transduced with 50MOI of the relevant hAd19a/64 vaccine and incubated for 5 h before the exchange to an FBS-free medium. About 20 h later, cells were lysed in RIPA Lysis Buffer (R0278; Thermo Scientific™, Waltham, MA, USA) with protease inhibitor (05892791001; Roche, Basel, Switzerland), in accordance with Thermo Scientific’s protocol. Cell lysates were denatured using NuPAGE™ LDS Sample Buffer (NP0007; Invitrogen™, Waltham, MA, USA) and NuPAGE™ Sample Reducing Agent (NP0004; Invitrogen™, Waltham, MA, USA) and heat-treated at 70 °C for 10 min in accordance with Invitrogen™ NuPAGE™ protocol. Hereafter, samples and a PageRuler™ Plus Prestained Protein ladder (26619; Thermo Scientific™, Waltham, MA, USA) were run on a NuPAGE™ Bis-Tris Mini Gel (NP0321; Invitrogen™, Waltham, MA, USA) with MOPS SDS Running Buffer containing NuPAGE™ Antioxidant (NP0005; Invitrogen™, Waltham, MA, USA). Proteins were transferred to an iBlot™2 nitrocellulose membrane (IB230002; Invitrogen™ Waltham, MA, USA) using an iBlot2 machine and blocked for 1 h with 5% skimmed milk in TBS-T. After washing, membranes were incubated overnight at 4 °C with a primary antibody, which was either anti-T2A-antibody (Crb200569d; CRB discoveries, Cleveland, UK; 1:2000) to detect P2A peptide on HERV-W Gag or a housekeeping control protein anti-GAPDH antibody (ab181602; Abcam, Cambridge, UK; 1:8000). Following this, membranes were incubated for 1 h at room temperature with a secondary polyclonal goat anti-rabbit IgG antibody (P0448; Dako, Glostrup, Denmark). Membranes were developed using LumiGlo Chemiluminescent (5430, KPL, LGC group, Teddington, UK) or SuperSignal West Femto Maximum Sensitivity Substrate (34095; Thermo Scientific™, Waltham, MA, USA) and imaged with an iBright CL1500. Relative expression differences of bands were analyzed with the iBright analysis software V5.1.0.

### 4.6. Transmission Electron Microscopy (TEM)

TEM was performed as described in [7] by the Core Facility for Integrated Microscopy at the University of Copenhagen. In brief, A549 cells were transduced with either 50MOI of hAd19a/64 vectored HERV-W ISDwt vaccine, MelARV ISDwt vaccine [7], or 20MOI of HERV-K ISDwt vaccine, similarly designed with Gag and Env consensus sequences separated by an antigen-presentation tag and P2A (unpublished, patent no. WO2019043127). The transduced A549 cells were cultured for 24 h on Thermanox coverslips (150067, Thermo Scientific™, Waltham, MA, USA) pre-incubated with poly-L-lysine. Cells were fixed with 2% glutaraldehyde in 0.05 M sodium phosphate buffer (pH 7.2). Specimens were rinsed with 0.15 M phosphate Buffer (pH 7.2) and postfixed in 1% OsO4 within 0.12 M sodium phosphate buffer (pH 7.2) for 2 h. Specimens were dehydrated in graded series of ethanol, transferred to propylene oxide, and embedded in Epon. Next, the Thermanox coverslips were removed, and sections of ~60 nm were cut with a Leica UC7 microtome (Leica Microsystems, Wetzlar, Germany). Finally, sections were collected on copper grids with Formvar supporting membranes and stained with uranyl acetate and lead citrate. Sections were examined using a Philips CM 100 Transmission EM (Philips, Amsterdam, The Netherlands), operated at an accelerating voltage of 80 kV, and images were captured with an OSIS Veleta digital slow scan 2k × 2k CCD camera and the ITEM software package.

### 4.7. Transduction of T24 Cells for Light Microscopy Pictures

T24 cells were either transduced with 12.5MOI of the relevant hAd19a/64 HERV-W vaccines or left untransduced. Cell morphology was assessed by light microscopy pictures of unstained and unfixed cells using the ZOE Cell Imager (BioRad, Hercules, CA, USA) 24 h post-transduction.

### 4.8. Transduction of Murine Bone-Marrow-Derived Dendritic Cells (BMDCs)

Culturing and maturation of murine BMDCs were based on a previously published study by Jin et al. [53]. Cells were flushed from the femur of BALB/c mice. Red blood cells were lysed using ACK lysing buffer (A1049201; Thermo Scientific™, Waltham, MA, USA), and the remaining cells were seeded at a concentration of 1 × 10^4^ cells/mL in 100 mm non-tissue treated Petri dishes with 10 mL RPMI 1640 Medium, Glutamax Supplement containing 10% FBS, 1% sodium pyruvate, 1% HEPES (1 M), 1% MEM non-essential amino acid solution (100×), and 55 µM 2-Mercaptoethanol (50 mM), and 20 ng/mL murine GM-CSF. The medium was exchanged completely on day 3, and half on day 6 and day 8. Floating cells were spun down and added back to the culture. On day 10, half of the medium was exchanged supplemented with 5 ng/mL of murine IL-4. On day 11 and day 12, three-quarters and the medium were exchanged, respectively, and supplemented with GM-CSF and IL-4. On day 13, for the last 20 h of culturing, all the medium was exchanged and supplemented with GM-CSF, IL-4, 0.5 µg/mL of CpG, and 1 µg/mL of LPS to fully mature the BMDCs. On day 14, the mature BMDCs were harvested using Versene and seeded in round-bottom 96-well plates at a concentration of 0.1 × 10^6^ cells per well in a medium containing GM-CSF and IL-4. Experimentally, a vaccine concentration of 1000MOI was found necessary for the transduction of murine BMDCs with the hAd19a/64 serotype, which is consistent with previously published literature of lower transduction efficacy with hAd19a/64 in murine cells [54].

### 4.9. Staining of Murine BMDCs

For evaluation of the surface expression of the HERV-W Env and cell surface activation markers of BMDCs, BMDCs were stained 24 h after transduction. The BMDCs were washed with FACS buffer and incubated with anti-mouse CD16/CD32 Fc-block (Clone 2.4G2, 553141; BD Biosciences, Franklin Lakes, NJ, USA) for 5 min on ice. Then, primary rabbit anti-human polyclonal HERV antibody (PA5-22819; Invitrogen™, Waltham, MA, USA) was added and incubated for 30 min at 4 °C. Cells were then incubated with the secondary PE donkey anti-rabbit IgG antibody (406421; BioLegend^®^, San Diego, CA, USA; 1:100) and eBioscience™ Fixable Viability Dye eFlour™ 780 (65-0865; Invitrogen™, Waltham, MA, USA; 1:1000) for 30 min at 4 °C. Hereafter, cells were stained for 20 min at 4 °C using a dilution 1:250 of the following fluorophore-conjugated antibodies: BV510 rat anti-mouse I-A/I-E (742893, BD Biosciences), APC hamster anti-mouse CD11c (561119; BD Biosciences, Franklin Lakes, NJ, USA), BV421 rat anti-mouse CD86 (105031; BioLegend^®^, San Diego, CA, USA), and PerCP/Cy5.5 rat anti-mouse CD40 (124623; BioLegend^®^, San Diego, CA, USA). Cells were fixated in 1% PFA for 15–20 min at 4 °C and run on an LSRFortessa™ 5-laser flow cytometer (BD Biosciences, Franklin Lakes, NJ, USA). The data were analysed using FlowJo™ v10 analysis software and GraphPad Prism 9. Expression differences were calculated using a two-tailed paired t-test with statistical significance levels defined as * = *p* < 0.05 and ** = *p* < 0.01.

### 4.10. BMDC Proinflammatory Biomarkers

Supernatants from BMDCs were isolated and frozen 24 h post-transduction. Concentrations of different biomarkers in the supernatant were measured in duplicates using a customized version of the V-PLEX mouse proinflammatory cytokine panel 1 kit (K15048D, Mesoscale, Rockville, MD, USA) at a 1:5 dilution, according to the instructions of the kit. Concentrations were analysed using the MESO QuickPlex SQ 120MM (Mesoscale, Rockville, MD, USA).

### 4.11. Generation of HERV-W Env Expressing RenCa Cell Line

The murine RenCa cells were transduced with 1 mg/mL sequabrene (Sirion Biotech, PerkinElmer, Germany) mixed with 30MOI of a lentivirus encoding HERV-W Env under the control of a CMV promoter. The lentivirus contained no selection markers and was provided by the company Sirion Biotech. Media was exchanged ~24 h after transduction. Four days after transduction, cells were detached with trypsin and stained with primary HERV-Env antibody and secondary PE-conjugated IgG-antibody to detect HERV-W Env, following the same protocol as for the transduced A549 cells. Live cells expressing HERV-W Env were bulk-sorted with a FACS ARIA-II (BD Biosciences, Franklin Lakes, NJ, USA). Positively sorted cells were seeded in a 6-well plate and cultured and expanded for freezing stocks.

### 4.12. Animal Procedures

Female BALB/c and C57BL/6 mice were obtained from Envigo (Scandinavia) at the age of 6–8 weeks and housed at the Panum Institute, University of Copenhagen. Mice were housed in ventilated cages of five mice per cage, and the environmental parameters were 8–10 air changes per hour, temperature: 22 °C (±2 °C), and humidity: 55% (±10%).

Mice were acclimatized for minimum one week prior to any experiments. All mice experiments were performed according to national guidelines, and experimental procedures were approved by the National Animal Experiments Inspectorate (Dyreforsøgstilsynet, license no. 2019-15-0201-00203).

Mice were anesthetized with isoflurane and vaccinated s.c. in the lower right limb with 30 µL of the relevant vaccine mixed with 1× PBS at 2 × 10^7^ infectious units. At the end of each experiment, mice were euthanized by cervical dislocation.

For the tumour challenge, HERV-W Env expressing RenCa cells were detached with trypsin, washed, counted, and resuspended in PBS at a concentration of 5 × 10^6^ cells/mL. Cells were kept on ice until injection. BALB/c mice were injected either s.c. in the right flank or i.v. in the tail vein with 100 µL cell suspension, 0.5 × 10^6^ cells per mouse. Mice subjected to the same injection route were randomized. Cell viability was measured pre- (82.5%) and post-injection (73.5%). Four days post tumour challenge, mice were vaccinated as described above (*n* = 10 per group). S.c. tumour growth was measured by calliper three times weekly, and the tumour volume was calculated using the following formula: length × width^2^ × 0.5236 [55]. Small palpable tumours that could not be measured with a calliper were set as 0.5236 mm^3^. Mice subjected to tumour cells i.v. were checked regularly until the first signs of sickness. Hereafter, mice were checked daily until the end of the experiment. Sick mice reaching human endpoints (starey coat, bent over position, reduced mobility) were euthanized by cervical dislocation. Lungs were collected and evaluated for the presence of tumour nodules. GraphPad Prism 9 was used to analyse statistical differences in survival between the groups with a log-rank (Mantel-Cox test) with a statistical significance level defined as * = *p* < 0.05.

### 4.13. Serum Isolation

Blood samples were taken from the cheek and stored overnight at 4 °C. Serum was isolated from the blood samples by two centrifugations at 800× *g* at 8 °C for 8 min.

### 4.14. Evaluation of HERV-W Env-Specific Antibody Responses

In a 96-well plate, 0.2 × 10^6^ RenCa and HERV-W Env positive RenCa cells were seeded per well and stained with pre-bleed and end-bleed serum from vaccination experiments in a 1:20 dilution for 1 h at 4 °C. Cells were stained with PE goat anti-mouse IgG (405307; BioLegend^®^, San Diego, CA, USA; 1:100) and eBioscience™ Fixable Viability Dye eFlour™ 780 (65-0865; Invitrogen™, Waltham, MA, USA; 1:1000) for 30 min at 4 °C. Cells were fixated in 1% PFA for 15 min at room temperature, and flow cytometry was performed on a LSRFortessa™ 3-laser flow cytometer (BD Biosciences, Franklin Lakes, NJ, USA). The data were analysed using FlowJo™ v10. In GraphPad Prism 9, response differences were calculated using the Mann–Whitney *t*-test with a statistical significance level defined as * = *p* < 0.05 and ** = *p* < 0.01.

### 4.15. Evaluation of IgG2a/IgG1 Antibody Ratio

The surface subunit of HERV-W Env (Syncytin-1), spanning from amino acid 21 to 313 of the Env sequence, was codon optimized for expression in *Trichoplusia ni* cells. The DNA construct containing a strep Tag II-tag at the C-terminal end was synthesized by Geneart (Regensburg, Germany) and subcloned into the baculovirus expression vector pAcGP67-A (BD Biosciences, Franklin Lakes, NJ, USA). The secreted recombinant protein was produced in X5 cells and purified on a streptactin HP column. Purity was assessed by SDS-PAGE.

Wells of Nunc MaxiSorp 96-well plates (442404; Thermo Scientific™, Waltham, MA, USA) were coated and incubated overnight at 4 °C with either 0.2 µg of the recombinant HERV-W Env surface subunit protein, or a four-fold serial dilution of IgG1 (401401, BioLegend^®^, San Diego, CA, USA) or IgG2a (401501, BioLegend^®^, San Diego, CA, USA) isotype antibody starting at 75 ng. The following day, wells were washed and then blocked with PBS containing 0.1% Tween-20 and 0.5% BSA for 1 h at room temperature. Hereafter, wells were washed and then incubated with relevant BALB/c mouse pre-bleed serum (day 0) (1:50 dilution) or end-bleed serum (day 14) (four-fold serial dilution, starting with 1:50) for 1 h at room temperature. As a positive control, rabbit anti-human polyclonal HERV antibody (PA5-22819; Invitrogen™, Waltham, MA, USA) was added in a three-fold serial dilution starting at 1µg/mL. After the incubation, wells were washed. Next, secondary anti-mouse IgG1/HRP (SBA-1071-05; SouthernBiotech, Birmingham, AL, USA; 1:8000) or IgG2a/HRP (SBA-1081-05; SouthernBiotech, Birmingham, AL, USA; 1:8000) antibody was added to cells with serum, whereas positive control wells were added secondary anti-rabbit IgG/HRP (P0448; Dako, Glostrup, Denmark, 1:2000). Wells were incubated for 1 h at room temperature and then washed. Finally, the antibodies were detected using TMB substrate and 1 M stop-solution H_2_SO_4_. Absorbance was measured at 450 nm using an ELISA plate reader, and GraphPad Prism 9 was used to analyse response differences.

### 4.16. Peptides

Using the NetMHC-4.0 online tool for BALB/c (H-2-Dd, H-2-Kd, H-2-Ld) and C57BL/6 (H-2-Db, H-2-Kb) [45,46], 8- and 9-mer peptides from HERV-W Env and Gag sequences were predicted. Peptides were produced by KareBay BioChem (Monmouth, NJ, USA) at 80% purity. Peptide pools were designed to cover the HERV-W Env sequence. These consisted of 16-mer or 20-mer peptides overlapping by 11 or 15 amino acids, respectively. These peptides were divided into pools corresponding to the surface subunit (61 × 16-mer peptides, 60 × 20-mer peptides), the ectodomain of the transmembrane subunit (26 × 16-mer peptides, 27 × 20-mer peptides), or the transmembrane and cytosolic domain (16 × 16-mer peptides, 15 × 20-mer peptides). Peptide pools were produced by ProteoGenix (Schiltigheim, France) at 80% purity.

### 4.17. Intracellular Stain of Splenocytes 

Following cervical dislocation, mouse spleens were mashed through a 70 µm sterile net to obtain a single-cell suspension. Cells were then counted and seeded in a round bottom 96-well plate with RPMI 1640 medium containing 10% FBS, 100 units/mL pen/strep (15140122; Thermo Scientific™, Waltham, MA, USA), 1 mM sodium pyruvate (11360070; Thermo Scientific™, Waltham, MA, USA), and 3 µM Monensin. Lyophilized peptides were reconstituted in DMSO to a concentration of 1 µg/µL and hereafter diluted in media to a final concentration of 1 ng/µL per well. Splenocytes were incubated for 5 h at 37 °C in a 5% CO_2_ incubator.

After the 5 h of incubation, cells were washed and stained with the following cell surface antibodies conjugated to fluorophores: BV421 rat anti-mouse CD8b antibody (126629; BioLegend^®^, San Diego, CA, USA), PE-Cy7 rat anti-mouse CD4 (561099; BD Biosciences, Franklin Lakes, NJ, USA), PerCP-Cy5.5 rat anti-mouse CD45R/B220 (552771, BD Biosciences), and FITC rat anti-mouse CD44 (553133; BD Biosciences, Franklin Lakes, NJ, USA). Hereafter, splenocytes were fixated in 1% PFA and permeabilized with Saponin before staining with two intracellular fluorophore-conjugated antibodies: APC rat anti-mouse IFNγ (554413; BD Biosciences, Franklin Lakes, NJ, USA); and PE rat anti-mouse TNFα (554419; BD Biosciences, Franklin Lakes, NJ, USA). Samples were run on the LSRFortessa-3 and analysed using the FlowJo™ v10 software. For percentage and total response numbers, responses of unstimulated samples were subtracted. Statistical differences between the two vaccine groups were calculated with the non-parametric Mann–Whitney test using the GraphPad Prism 9 software. Flow run failures and values below zero were excluded. Statistical significance levels are defined as * = *p* < 0.05 and ** = *p* < 0.01.

## 5. Conclusions

An increasing number of studies find HERV-W expression in cancers, and although the role of HERV-W in tumourigenesis is still poorly understood, the enriched expression in cancer makes it a potential target for therapies, provided that these therapies are capable of discriminating between self- and non-self HERV-W expression levels. Regulated T-cell receptor or antibody-based cell therapy as well as vaccines may realize this potential. In this study, we developed an anti-cancer vaccine candidate targeting HERV-W, based on a hAd19a/64-vector encoding the HERV-W Env, Syncytin-1, and an assembled sequence of HERV-W Gag. We identified two MHC-I peptides and peptide pools for measuring T-cell responses in mice. Additionally, we created a murine HERV-W Env-expressing tumour cell line for use as a distant tumour model in BALB/c mice. Our HERV-W ISDwt vaccine generated adaptive immune responses in inbred mice, and, in support of the “ISD hypothesis”, we found the ISDwt version more immunogenic than the ISDrev counterpart. Importantly, the HERV-W ISDwt vaccine-induced immune responses were sufficient to increase the probability of survival to HERV-W Env-positive tumours in mice. These results lay the groundwork for developing a cancer vaccine to target HERV-W-positive cancers in humans.

## Data Availability

Data is contained within the article or the Supplementary Material. The raw data is available upon reasonable request.

## Supplementary Materials

The supporting information can be downloaded at: https://www.mdpi.com/article/10.3390/ijms24129972/s1.

## Abbreviations

APC	Antigen-presenting cells
BMDC	Bone-marrow-derived dendritic cells
CT	Cytoplasmic domain
Ecto	Ectodomain
Env	Envelope
F-MuLV	Friend murine leukaemia virus
Gag	Group-specific antigen
hAd19a/64	Human adenovirus type 19a/64
HERV	Human endogenous retrovirus
HIV-1	Human immunodeficiency virus type 1
ICI	Immune checkpoint inhibitors
ICS	Intracellular staining
ISD	Immunosuppressive domain
ISDmut	Immunosuppressive domain mutated
ISDrev	Immunosuppressive domain reversed
ISDwt	Immunosuppressive domain wild-type
i.v.	Intravenous
mCAT1	Murine cationic amino acid transporter-1
MelARV	Melanoma associated retrovirus
MFI	Geometric mean fluorescent intensity
MHC-I/MHC-II	MHC class I/MHC class II
MOI	Multiplicity of infection
Neg. ctrl	Negative control
PE	Phycoerythrin
Pen/strep	Penicillin-streptomycin
PFA	Paraformaldehyde
s.c.	Subcutaneous
SU	Surface subunit
TEM	Transmission electron microscopy
TM	Transmembrane domain
TU	Transmembrane subunit
VLP	Virus-like particle
VLV	Virus-like vaccine
