# Supplementary material for: Human Ad19a/64 HERV-W Vaccines Uncover Immunosuppression Domain-Dependent T-Cell Response Differences in Inbred Mice
Source: Int J Mol Sci. 2023 Jun 9;24(12):9972. doi: 10.3390/ijms24129972 (PMC10297909; doi:10.3390/ijms24129972)
Supplement: Supplementary file 1 [file ijms-24-09972-s001.zip › ijms-2430239-supplementary.pdf]

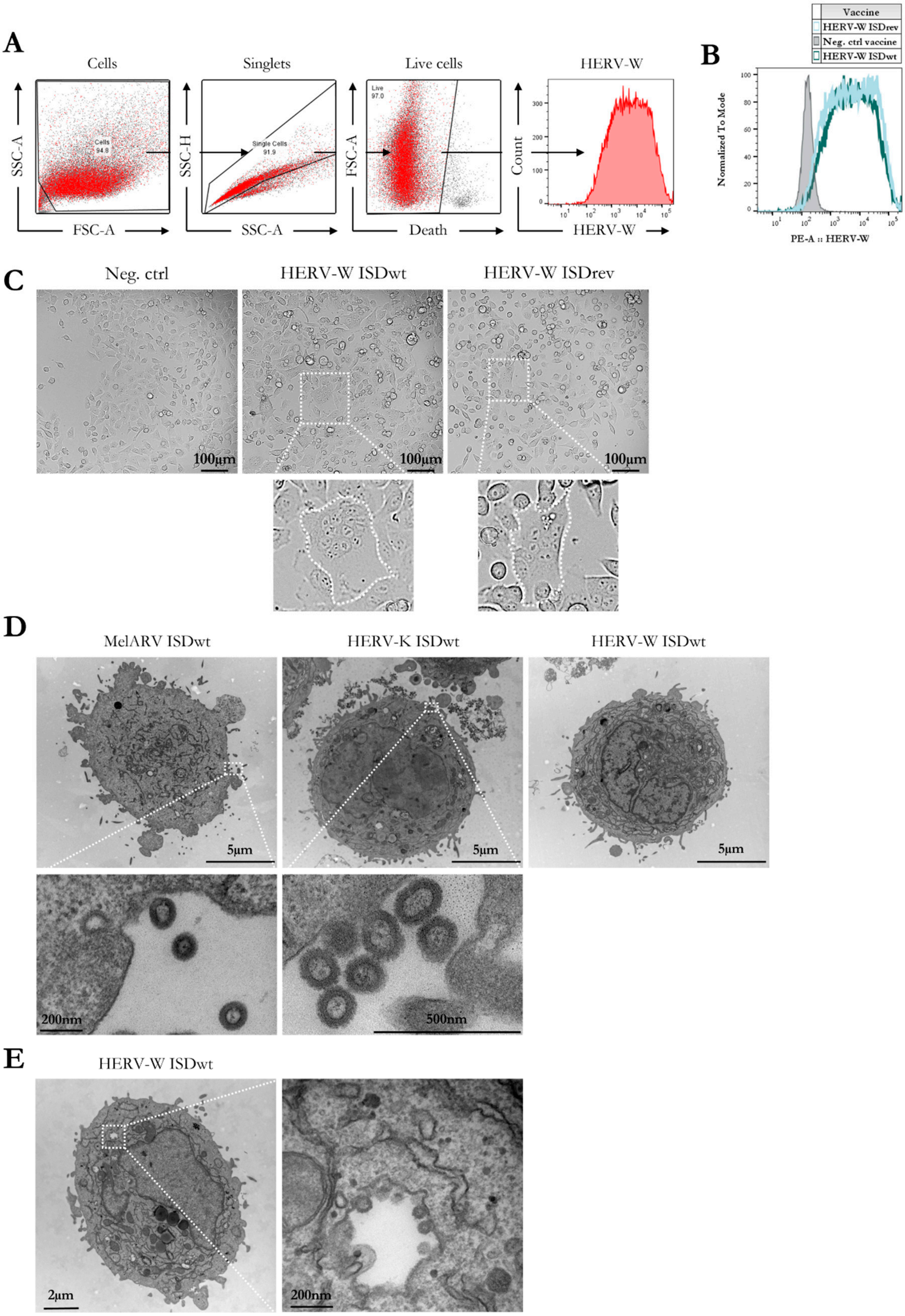
A
Cells
Singlets
Live cells
HERV-W
SSC-A
FSC-A
SSC-H
SSC-A
FSC-A
Death
Count
HERV-W
Cells 94.8
Single Cells 91.9
Live 97.0
B
Vaccine
HERV-W ISDrev
Neg. ctrl vaccine
HERV-W ISDwt
Normalized To Mode
PE-A :: HERV-W
C
Neg. ctrl
HERV-W ISDwt
HERV-W ISDrev
100μm
100μm
100μm
D
MelARV ISDwt
HERV-K ISDwt
HERV-W ISDwt
5μm
5μm
5μm
200nm
500nm
E
HERV-W ISDwt
2μm
200nm

Supplementary Figure s1. (A) Gating strategy for flow cytometry analysis of HERV-W Env expression levels on the surface A549 cells 24h after transduction. **(B)** Histogram overlay of HERV-W Env surface expression of A549 cells, 24h after transduction with either hAd19a/64 vectored HERV-W ISDwt, HERV-W ISDrev, or empty vaccine (Neg. ctrl vaccine). **(C)** Representative light microscopy of fused human T24 cells 24h post transduction with 12.5MOI of HERV-W ISDwt or HERV-W ISDrev vaccine compared to no transduction, using 20x objective. The lower panel shows mega cells resulting from the fusion of several cells. **(D)** Transmission electron microscopy (TEM) pictures of A549 cells transduced with 50MOI of hAd19a/64-MelARV ISDwt or HERV-W ISDwt, or 20MOI of HERV-K ISDwt. The lower panel shows virus-like particles (VLPs) released from the transduced cells. **(E)** TEM pictures of A549 cells infected with 50MOI of HERV-W ISDwt and zoom-ins on VLP-like structures in the cytosol (left) and vesicles (right) of infected cells.

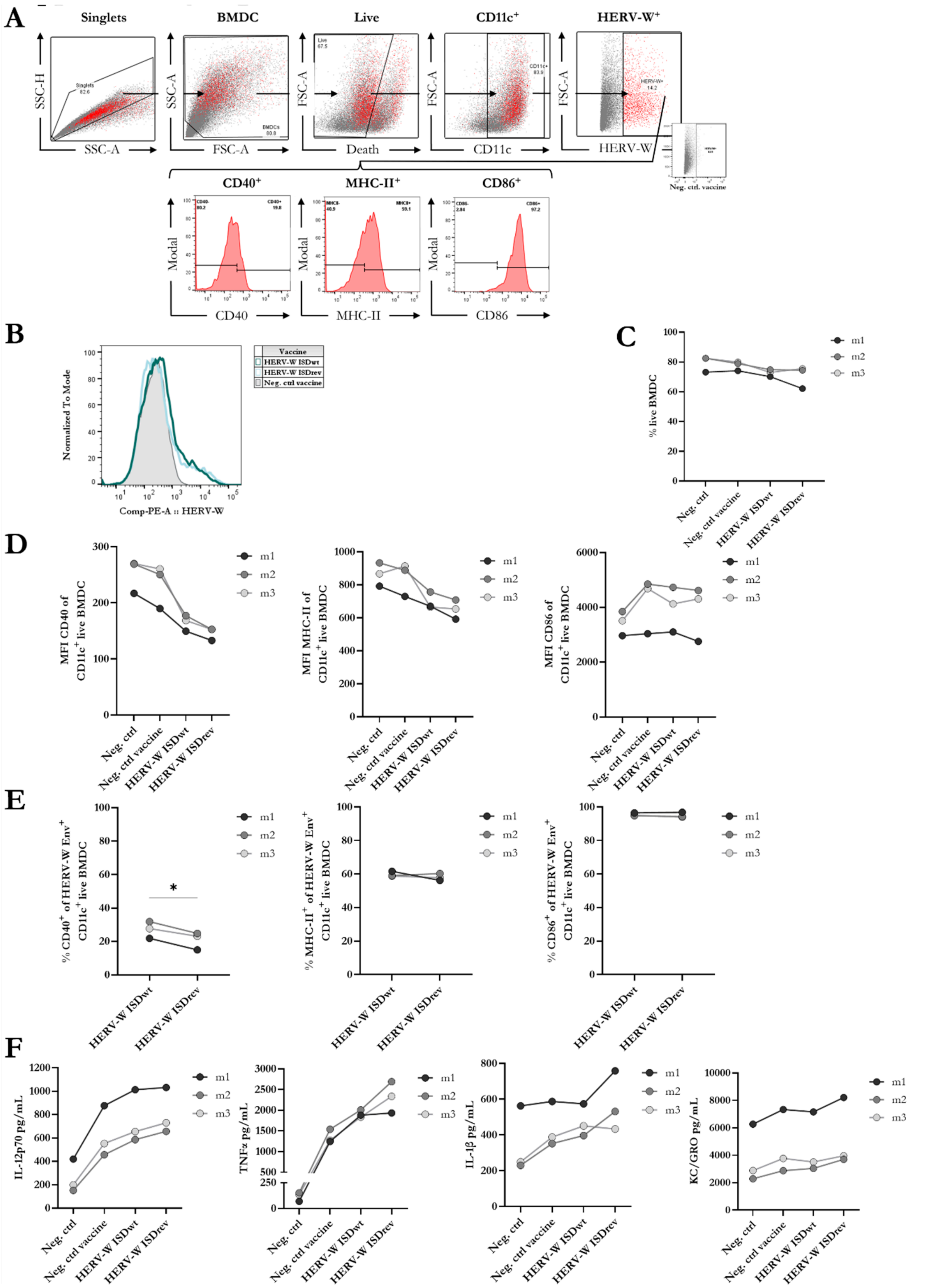

Supplementary Figure s2. Flow cytometry of matured bone-marrow-derived dendritic cells (BMDCs) isolated from BALB/c mice (*n* = 3) and transduced with either the empty hAd19a/64 vector (Neg. ctrl vaccine), the HERV-W ISDwt vaccine, or the HERV-W ISDrev vaccine at 1000MOI, 24h prior to analysis.

(A) Gating strategy for flow cytometry analysis of activation markers and HERV-W Env cell surface expression on BMDCs. Fluorescent minus one (FMO) samples and the Neg. ctrl vaccine were used to set the gates for activation markers and HERV-W Env expression. **(B)** Graph illustrating the expression of HERV-W Env on the surface of transduced BMDCs. **(C)** Percentage of live BMDCs following transduction. **(D)** Geometric mean fluorescent intensity (MFI) of either CD40, MHC-II, or CD86 (from left to right graph) out of $CD11c^+$ live BMDCs. **(E)** Percentage of either CD40, MHC-II, or CD86 (from left to right graph) out of HERV-W Env^+ $CD11c^+$ live BMDCs for each BALB/c mouse, analysed by flow cytometry. Expression differences were calculated using a two-tailed paired t-test and statistical significance is indicated with asterisks; * = $p<0.5$. Bullets depict the mean of three technical replicates per mouse (m1-3). Lines link BMDCs transduced or untransduced from the same mouse. **(F)** Concentrations of proinflammatory biomarkers (IL-12p70, TNFα, IL-1β, KC/GRO) from the supernatant of BMDCs 24h after transduction. Bullets illustrate the mean of two technical measurements from each mouse.

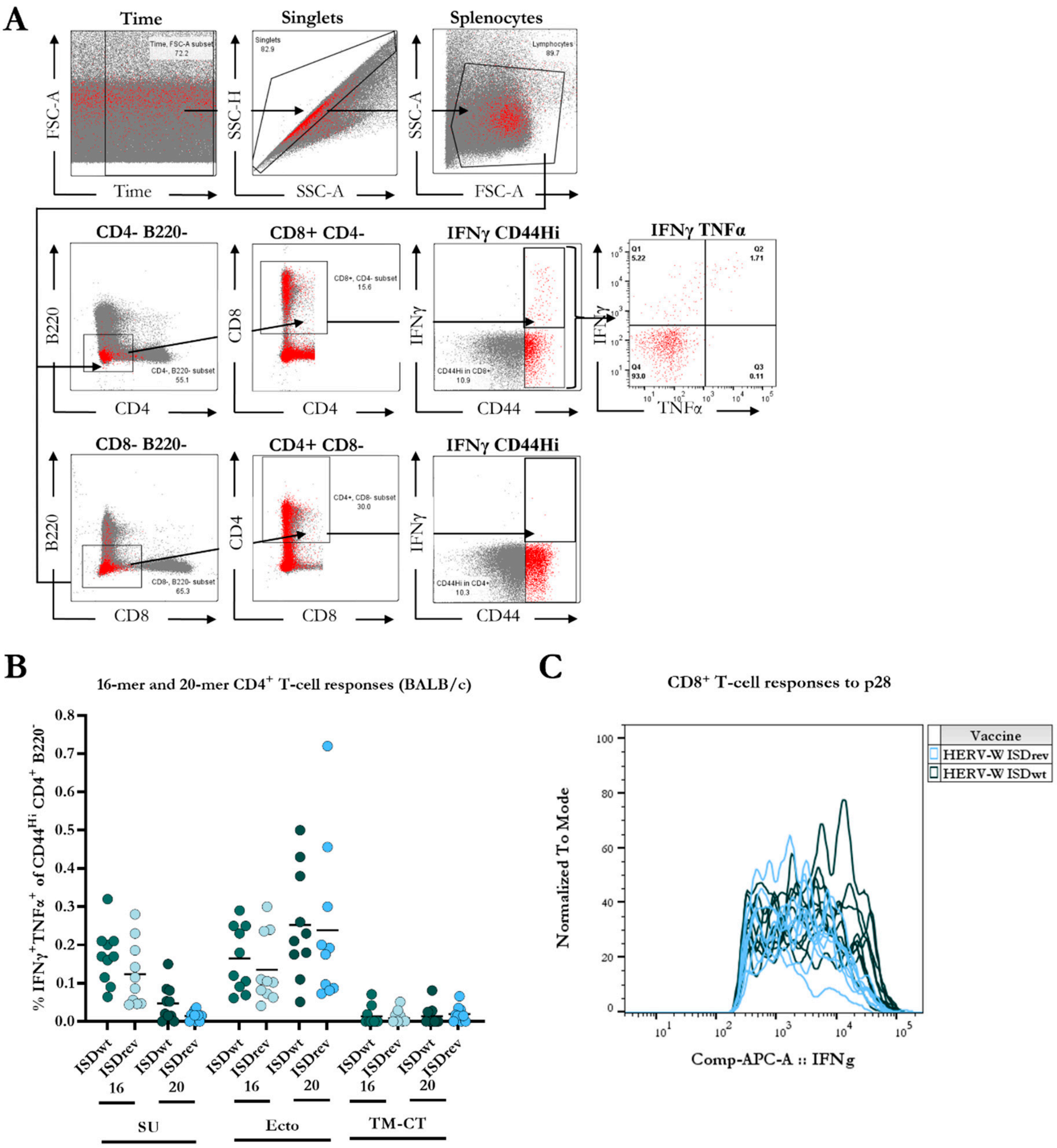
A
Time
Singlets
Splenocytes
Time, FSC-A subset
72.2
Singlets
82.9
Lymphocytes
89.7
FSC-A
Time
SSC-H
SSC-A
SSC-A
FSC-A
CD4- B220-
CD8+ CD4-
IFNγ CD44Hi
IFNγ TNFα
CD4-, B220- subset
55.1
CD8+, CD4- subset
15.6
CD44Hi in CD8+
10.9
Q1
5.22
Q2
1.71
Q4
93.0
Q3
0.11
B220
CD4
CD8
CD4
IFNγ
CD44
IFNγ
TNFα
CD8- B220-
CD4+ CD8-
IFNγ CD44Hi
CD8-, B220- subset
65.3
CD4+, CD8- subset
30.0
CD44Hi in CD4+
10.3
B220
CD8
CD4
CD8
IFNγ
CD44
B
16-mer and 20-mer CD4+ T-cell responses (BALB/c)
% IFNγ+TNFα+ of CD44Hi CD4+ B220-
ISDwt
ISDrev
16
20
SU
Ecto
TM-CT
C
CD8+ T-cell responses to p28
Normalized To Mode
Comp-APC-A :: IFNg
Vaccine
HERV-W ISDrev
HERV-W ISDwt

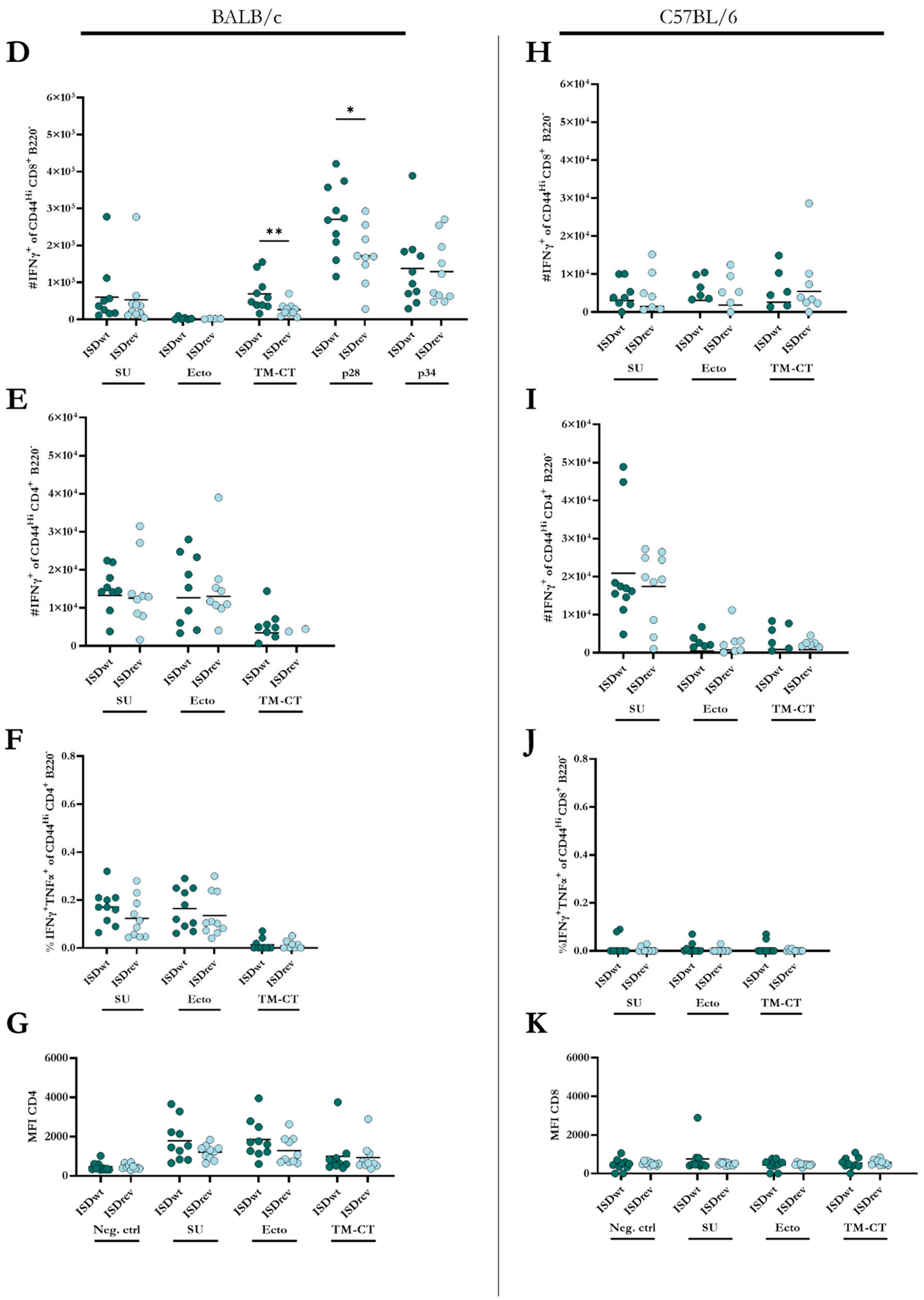

Supplementary Figure s3. (A) Gating strategy for flow cytometry analysis of intracellular staining (ICS), showing the population of IFNγ-and TNFα-producing CD8+ and CD4+ T-cells from spleens of vaccinated mice. **(B)** Comparison of the percentage of IFNγ-and TNFα-responding CD4+ T-cells to stimulation with 16-mer and 20-mer peptide pools in BALB/c (*n* = 10) vaccinated with either HERV-W ISDwt or HERV-W ISDrev vaccines, 14 days prior to analysis. **(C)** Histogram overlay of the geometric mean fluorescent

intensity (MFI) of IFNγ$^+$ out of IFNγ$^+$, CD44^{Hi}, CD8$^+$ B220$^-$ cells, responding to peptide 28 (p28) measured in BALB/c vaccinated with either of the two HERV-W vaccines. **(D-K)** BALB/c (*n* = 10) and C57BL/6 (*n* = 10) were immunized subcutaneously (s.c.) with either HERV-W ISDwt or HERV-W ISDrev. Splenocytes were isolated 14 days later and stimulated with single peptides or peptide pools (highlighted in Figure 3A) to measure IFNγ and TNFα producing T-cells. The total number of IFNγ responding CD8$^+$ and CD4$^+$ T-cells, calculated back to the number of lymphocytes of the entire spleen from BALB/c mice **(D-E)** and C57BL/6 mice **(H-I)**. Percentage of IFNγ and TNFα responding CD4$^+$ T-cells from BALB/c mice **(F)** and CD8$^+$ T-cells from C57BL/6 **(J)**. MFI of IFNγ and TNFα responses in CD4$^+$ T-cells from BALB/c mice **(G)** and CD8$^+$ T-cells from C57BL/6 mice **(K)**. Response differences were calculated by a non-parametric, two-tailed Mann-Whitney test, and significance levels are indicated by asterisks; * = $p<0.05$ and ** = $p<0.01$.

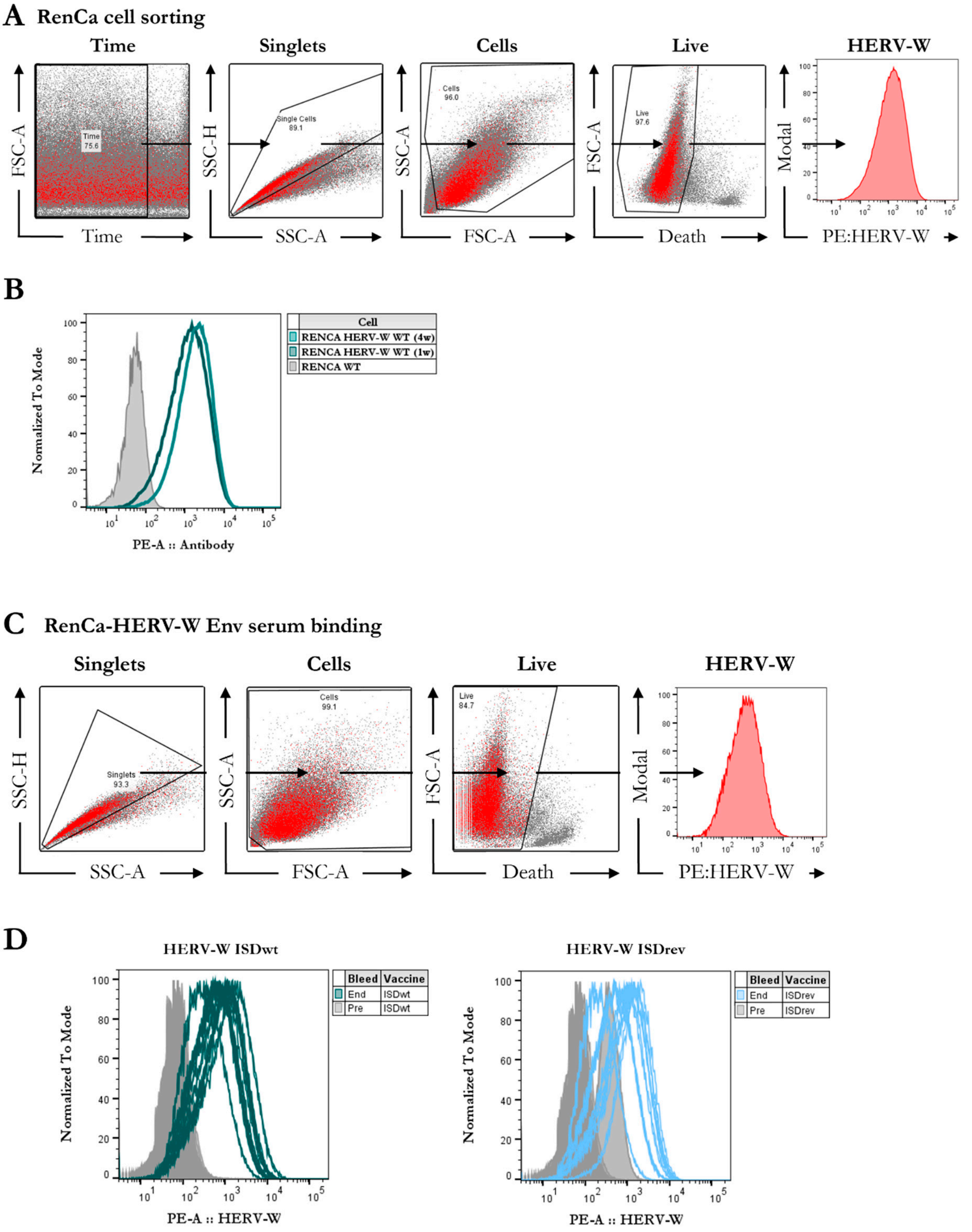

Supplementary Figure s4. (A) Gating strategy from cell sorting of RenCa cells transduced with HERV-W Env-encoded lentivirus. **(B)** Histogram overlays showing the expression stability of HERV-W Env protein on the surface of transduced RenCa cells. The expression was measured by flow cytometry at four weeks after sorting (light green) and one week from the same cell line frozen and re-cultured (dark green). **(C)** Gating strategy showing serum binding to HERV-W Env$^+$ RenCa cells. **(D)** Histograms overlays showing serum binding to HERV-W Env$^+$ RenCa cells pre-bleed (before vaccine "Pre") and end-bleed ("End") of BALB/c mice vaccinated with either the HERV-W ISDwt vaccine (left panel) or HERV-W ISDrev vaccine (right panel).
